# Micro‐Needling Fractional Radiofrequency Therapy for Facial Excoriation Disorder

**DOI:** 10.1111/jocd.70505

**Published:** 2025-10-23

**Authors:** Yuxiao Shao, Heng Zhang

**Affiliations:** ^1^ Department of Medical Cosmetology Yancheng TCM Hospital Affiliated to Nanjing University of Chinese Medicine Yancheng Jiangsu China

**Keywords:** acne vulgaris, excoriation disorder, micro‐needling fractional radiofrequency, skin picking

Dear Editor,

A 28‐year‐old female presented with a 3‐year history of habitual skin picking, initially triggered by the development of acne vulgaris (AV) on her face. She reported being unaware of using her fingernails to excoriate and compress lesions, persisting until pruritus, pain, or bleeding occurred. She would pick at healing scabs, denudating the skin surface. The patient picks for about 2 to 3 h daily, despite repeated attempts to stop or reduce skin picking; she demonstrated an inability to resist the urge. Skin picking led to discomfort, and the presence of post‐inflammatory hyperpigmentation (PIH) and facial scars caused her embarrassment, resulting in reluctance to make eye contact. She reported frequent escalation of skin picking behaviors during anxiety states. The patient had an unremarkable past medical history. Physical examination revealed facial PIH and scarring, with focal areas showing ulcerations, crusted lesions, and sparse inflammatory papules and pustules (Figure 1a,c,e). Complete blood count, fasting glucose, sex hormone panel, and thyroid function tests were within normal limits. These findings are consistent with excoriation disorder (ED). The patient was administered two micro‐needling fractional radiofrequency (MFR) treatment sessions 2 months apart. The Skin Picking Scale‐Revised (SPS‐R) scores of the patient decreased from 13 to 9, concomitant with clinical improvement in dermatological manifestations (Figure 1b,d,f) at the 12‐week follow‐up. We are still conducting the follow‐up for this patient.

**FIGURE 1:**
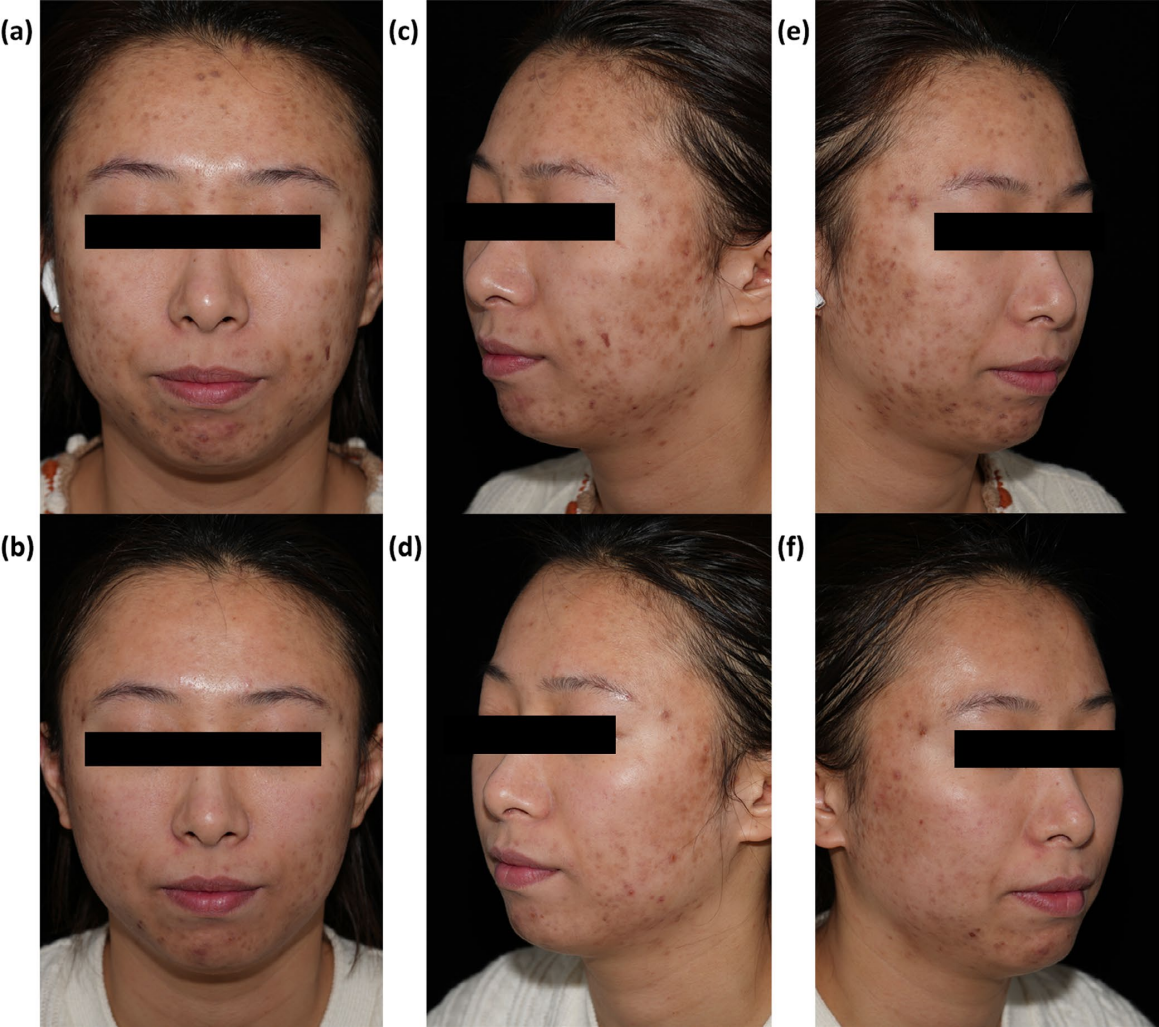
(a, c, e) The frontal, left lateral, and right lateral views of the patient before the treatment, with diffuse facial PIH and stellate scarring, focal areas showing ulcerations, crusted lesions, and sparse inflammatory papules and pustules. (b, d, f) Posttreatment outcomes after two sessions of MFR, comparative clinical photography at 12‐week follow‐up demonstrates significant improvement: Significant lightening of PIH was observed, with resolution of crusted lesions and excoriations, alongside partial resolution of inflammatory papules and pustules.

ED is a chronic psychogenic disorder characterized by compulsive recurrent skin picking, squeezing, or digging behaviors that result in tissue damage [1]. AV is one of the commonest precipitating factors for ED [1] and shows a rising incidence rate among adolescents and young adults. Due to symptomatic parallels with obsessive‐compulsive disorders (OCD), ED was classified under obsessive‐compulsive and related disorders in the DSM‐5 [2]. Our case fulfills the DSM‐5 criteria for diagnosis of ED: recurrent skin picking, resulting in skin lesions; repeated attempts to decrease or stop skin picking; the skin picking causes clinically significant distress or impairment in social, occupational, or other important areas of functioning; the skin picking cannot be attributed to the physiologic effects of substance abuse or another medical condition; the skin picking cannot be better explained by the symptoms of another mental health disorder. We used the SPS‐R to assess symptom severity and functional impairment, which can measure these constructs reliably, demonstrating high internal consistency [3]. ED triggered by AV is frequently missed in clinical practice, necessitating integrated assessment to detect underlying mental health pathologies [1]. These patients face compounded psychological burdens from both AV and ED, requiring simultaneous treatment of active acne inflammation and the emotional and social impacts linked to these conditions. In this reported case, where facial manifestations were dominated by scarring and PIH, we treated with MFR therapy, which concomitantly achieved enhancing the condition of acne through dermal remodeling and significant skin tightening effects. We observed a significant reduction in skin‐picking behaviors following MFR treatment, prompting the hypothesis that MFR may modulate cutaneous neurosensory pathways to restore normative tactile perception, thereby reducing compulsive urges. This mechanism warrants further investigation through rigorous neurophysiological studies.

The current paucity of literature on AV‐associated ED necessitates enhanced clinical awareness among dermatologists, esthetic physicians, and pediatricians regarding skin‐picking disorder diagnostics, coupled with established psychiatric referral pathways for severe cases [4]. In conclusion, a comprehensive and nonjudgmental approach to the management of ED in the target population is indispensable [1].

## Author Contributions

Yuxiao Shao composed the manuscript and provided clinical figures. Heng Zhang was involved in the acquisition, analysis, or interpretation of data for the work. All authors collaborated closely in the preparation of the final manuscript. They gave their final approval of the version to be published and agreed to be accountable for all aspects of the work.

## Ethics Statement

Reviewed and approved by Yancheng TCM Hospital Affiliated to Nanjing University of Chinese Medicine, approval # KY250701.

## Consent

The authors certify that the patient has given her informed consent for case details and images to be published. Institutional review board approval was obtained for this case study, ensuring compliance with ethical guidelines and patient confidentiality.

## Conflicts of Interest

The authors declare no conflicts of interest.

## Data Availability

The data that support the findings of this study are available from the corresponding author upon reasonable request.
